# Early interventions reduce multimorbidity and TB disability in Kenya, Uganda, Zambia, and Zimbabwe

**DOI:** 10.5588/ijtldopen.25.0656

**Published:** 2026-03-13

**Authors:** F.M. Banda, A. Bloom, J. Chakaya, R. Chimzizi, C. Chitalu, C. Duri, A.D. Harries, N. Kasese-Chanda, I. Kathure, F.N. Kavenga, A.M.V. Kumar, H. Luzze, I. Mbithi, M. Mputu, A. Mubanga, D. Mudoola, D. Nair, M. Ngwenya, S. Ntambi, P. Thekkur, C. Timire, E. Tweyongyere, M. YaDiul, R. Zachariah, The Kenya, Uganda, Zambia and Zimbabwe TB Disability Study Group

**Affiliations:** 1 University Teaching Hospital, Ministry of Health, Lusaka, Zambia;; 2 Public Health Specialist, Washington, DC, USA;; 3 Department of Medicine, Therapeutics, Dermatology and Psychiatry, Kenyatta University, Nairobi, Kenya;; 4 Respiratory Society of Kenya, Nairobi, Kenya;; 5 National Tuberculosis and Leprosy Programme, Ministry of Health, Lusaka, Zambia;; 6 Directorate of Health Services, Harare City Council, Harare, Zimbabwe;; 7 International Union Against Tuberculosis and Lung Disease, Paris, France;; 8 Department of Clinical Research, Faculty of Infectious and Tropical Diseases, London School of Hygiene and Tropical Medicine, London, UK;; 9 Division of National TB, Leprosy and Lung Disease Programme, Ministry of Health, Nairobi, Kenya;; 10 Ministry of Health and Child Care, AIDS and TB Department, Harare, Zimbabwe;; 11 The Union South-East Asia Office, New Delhi, India;; 12 Yenepoya Medical College, Yenepoya (Deemed to be University), Mangalore, India;; 13 National Tuberculosis and Leprosy Programme, Ministry of Health, Kampala, Uganda;; 14 Independent Researcher, Public Health Specialist, Scottsdale, Arizona;; 15 Mulago National Referral Hospital, Kampala, Uganda;; 16 UNICEF/UNDP/World Bank/WHO Special Programme for Research and Training in Tropical Diseases (TDR), World Health Organization, Geneva, Switzerland.

**Keywords:** tuberculosis, TB-associated disability, fourth 90, patient-centred holistic care, sub-Saharan Africa, real-time operational research, SORT IT, universal health coverage

## Abstract

**BACKGROUND:**

We provided early screening and referrals for multimorbidity and absenteeism and assessed changes in these parameters from TB treatment start to completion in health facilities across Kenya, Uganda, Zambia, and Zimbabwe.

**METHODS:**

A cohort study in 26 health facilities within national TB programmes.

**RESULTS:**

Follow-up was conducted in 1,146 (77%) of 1,497 patients; assessments took a median time of 30 min (interquartile range: 24–36). Symptom prevalence declined from 96% to 9%. Comorbidities (HIV, diabetes/hyperglycaemia, hypertension/high blood pressure) remained stable, while mental health disorders/probable depression decreased from 7% to 4%. Multimorbidity fell markedly; disability (inability to walk 400 m in 6 min) decreased from 20% to 4%; and those with ≥3 multimorbidity conditions decreased from 20% to 8%. Work/school absenteeism declined from 73% to 10%. Overall, referrals for care exceeded 85%, except for silica exposure (23%), smoking (57%), and recreational drug use (46%). Within-facility referrals were nearly 100%, except for silica exposure and disability (∼35%).

**CONCLUSION:**

Early interventions for multimorbidity led to major reductions in symptoms, risk factors, disability, and absenteeism, advocating for integration of patient-centred care throughout the TB care pathway. This multi-country study provides a promising roadmap for progress towards achieving this goal.

A growing body of evidence indicates that a substantial proportion of patients who successfully complete TB treatment continue to suffer comorbidities, risk factors, and disability (multimorbidity).^1–4^ These not only diminish quality of life but probably also contribute to the higher mortality observed amongst TB survivors as compared with the general population.^5^ Efforts are underway to address these challenges. Standards and guidelines have been proposed to support the assessment, management, and rehabilitation of patients with post-TB disease.^6^ The WHO has also published a policy brief on TB-associated disability, advocating for integrated care, prevention, and early detection of impairments (including disability) through systematic screening and timely referral.^7,8^ While this important policy, advocacy, and scientific work progresses, national TB programmes (NTPs) need to take up the mantle and act. Recent operational research from NTPs in China^9,10^ and East and Southern Africa^11^ have demonstrated that assessing patients for disability, comorbidities, and TB-related risk factors at the end of TB treatment and referring those in need for further care is both feasible and valuable.

We have argued that conducting these assessments and referrals at the end of TB treatment is too late; they should begin early, immediately after the patient has been started on TB treatment. Early interventions may improve TB treatment outcomes, lower the risk of recurrent TB, mitigate pulmonary disability (through early pulmonary rehabilitation), and decrease the risk of long-term morbidity and mortality.^12^ However, there is limited evidence on how well this works in the programmatic setting. Of two studies from China, one assessed risk factors and multimorbidity only at the end of TB treatment,^9^ while the other assessed the same parameters at both the start and completion of TB treatment.^10^ The latter study found that multimorbidity was less frequent or had improved following early interventions. While these results are promising, more evidence from other countries and settings is needed to draw widely applicable conclusions.

In 2024, we enrolled cohorts of TB patients through the NTPs of Kenya, Uganda, Zambia, and Zimbabwe. At the start of treatment, we assessed for multimorbidity and referred those in need of further care and support.^13^ We also assessed for work and school absenteeism associated with TB. In the current study, we reassessed the burden of multimorbidity and work absenteeism at the end of TB treatment to determine if this had changed compared with the start of treatment.

## METHODS

This was a cohort study conducted as real-time operational research within NTP services in four African countries. The study implemented a programmatic intervention that included training on screening for multimorbidities, referral, and linkage to care, followed by supervision and real-time monitoring throughout the course of TB treatment.

### Study sites and setting

The study was conducted in the 26 health facilities included in the previous cohort study assessing multimorbidity at the start of TB treatment in Kenya, Uganda, Zambia, and Zimbabwe.^13^ All facilities were located in and around the capital cities and were selected based on predefined criteria, including a sufficient caseload of TB patients and the capacity to provide follow-up care throughout the treatment period.

### Study population

The study population comprised consecutive patients aged ≥18 years initiating treatment for any form of TB, including drug-resistant disease between June and December 2024.

### Training of health professionals using an adapted SORT IT

As previously described, health facility staff were trained in May 2024 using an adapted SORT IT (Structured Operational Research Training Initiative) workshop focused on real-time operational research.^13–15^ Designated focal persons (including doctors, nurses, clinical officers, nutritionists, and community health promoters) were trained to conduct patient assessments and referrals and to assess work absenteeism at the end of TB treatment using the same methodology as at the start of treatment. At study conclusion, a face-to-face SORT IT workshop for investigators was convened in September 2025 to analyse the data and to collaboratively prepare this manuscript along with dissemination tools for stakeholders.

### Assessments for multimorbidity and work absenteeism

Focal persons assessed TB patients for multimorbidity and work absenteeism using the same methodology from the previous study.^13^ Comorbidities included HIV infection, diabetes mellitus (DM), high blood pressure, and mental health disorders. Patients were asked about prior diagnosis of these conditions. HIV status was obtained from the TB register or determined through testing if HIV status was negative or unknown. All patients underwent random blood glucose (RBG) testing, followed by fasting blood glucose (FBG), if indicated. Hyperglycaemia was defined as an FBG ≥7.0 mmol/L (≥126 mg/dL) or an RBG ≥11.1 mmol/L (≥200 mg/dL)^16^: patients meeting this criteria were referred to a DM or general medical clinic for diagnostic confirmation of DM. High blood pressure was defined as systolic blood pressure ≥140 mmHg and/or diastolic blood pressure ≥90 mmHg.^17^ Patients were screened for probable depression using the Patient Health Questionnaire (PHQ-2)^18^: patients scoring ≥3 were referred to a counsellor or a mental health specialist. TB-related risk factors included tobacco smoking (smoking at least once in the last 1 month), probable alcohol dependence (based on a Cut, Annoyed, Guilty, Eye [CAGE] questionnaire score ≥2),^19^ occupational silica exposure, recreational drug use, and undernutrition. Undernutrition was defined as a body mass index (BMI) < 18.5 kg/m^2^, calculated by measured height and weight. Disability was assessed using the 6-minute walking test (6MWT), a proxy for cardio-pulmonary function (aerobic capacity and endurance).^20^ Patients were instructed to walk back and forth along a measured track for 6 min with disability defined as walking <400 m in that time.^21^ Work absenteeism was evaluated through questions related to employment. The time taken to complete all these assessments, including the 6MWT, was recorded in minutes.

### Onward referrals for eligible patients

Focal persons at the health facilities provided advice and referred patients with multimorbidity to appropriate services within the same facility or, when necessary, to another health facility in the catchment area. Referral criteria were consistent with those used in the previous baseline study.^13^

### Data collection, analysis, and statistics

Individual patient data were collected using a pre-designed form, entered using EpiCollect5 and cross-checked by focal persons at the health facilities. The process was supervised by study coordinators. Data quality check reports were generated each week by PT and DN at the Centre for Operational Research, The Union. Data were analysed using STATA® (version 16.0 StataCorp LLC) and summarised using frequencies and proportions. The χ² test was used to assess differences in proportions of categorical variables. Univariate and multivariate generalised linear (modified Poisson regression) models were used to assess factors associated with disability at the end of the treatment. In the multivariate model, variables with *P* value < 0.05 in the univariate model were included. Crude and adjusted prevalence ratios (aPRs) with 95% confidence intervals (CIs) were reported.

### Ethical statement

Administrative approval was received from each NTP and ethics approvals from: the Union Ethics Advisory Group (EAG 01/24; 17/01/2024); the Kenya Medical Research Institute Scientific and Ethics Review Unit (KEMRI/RD/22; 20/05/2024); Mulago Research Ethics Committee (MHREC 2024-154; 21/06/2024); University of Zambia Biomedical Research Committee (5316-2024; 28/05/2024); and the Medical Research Council of Zimbabwe (MRCZ/A/3194; 19/06/2024). Study participants gave informed consent.

## RESULTS

Of 1,683 patients (28% female), who started TB treatment, 53 (3%) were lost to follow-up, 52 (3%) died, 14 (1%) failed treatment, 3 (<1%) switched treatment regimen, and 64 (4%) were not evaluated. Of the remaining 1,497 (89%) who successfully completed treatment, 1,146 (77%) were assessed for multimorbidity (Table 1).

**Table 1.** Demographic and clinical characteristics of TB patients aged ≥18 years assessed for multimorbidity^A^ at the end of successful TB treatment in selected health facilities in four African countries, June 2024 to September 2025.

Characteristics	Started treatment	Successfully completed treatment^B^	Assessed at end of treatment	*P* value
	n	n (%)^C^	n (%)^D^	
Total	1,683	1,497 (89)	1,146 (77)	
Country of recruitment				
Kenya	501	394 (79)	387 (98)	<0.001
Uganda	488	482 (99)	349 (72)	
Zambia	477	426 (89)	224 (53)	
Zimbabwe	217	195 (90)	186 (95)	
Age (in years)				
18–29	494	446 (90)	352 (79)	0.51
30–44	782	697 (89)	524 (75)	
45–59	330	290 (88)	220 (76)	
≥60	77	64 (83)	50 (78)	
Sex				
Male	1,204	1,072 (89)	811 (76)	0.19
Female	479	425 (89)	335 (79)	
Living area				
Urban	1,646	1,462 (89)	1,121 (77)	0.47
Rural	37	35 (95)	25 (71)	
Site of TB				
Pulmonary	1,607	1,438 (89)	1,094 (76)	0.03
Extra-pulmonary	76	59 (78)	52 (88)	
Type of TB				
Bacteriologically confirmed	1,047	950 (91)	734 (77)	0.39
Clinically diagnosed	636	547 (86)	412 (75)	
Category of TB				
New	1,510	1,351 (89)	1,028 (76)	0.20
Previously treated	173	146 (84)	118 (81)	
Drug-susceptibility				
Sensitive	1,657	1,472 (89)	1,130 (77)	0.14
RR/MDR TB	26	25 (96)	16 (64)	

RR/MDR TB = rifampicin-resistant/multidrug-resistant TB.

A Multimorbidity = comorbidities, risk factors, and disability.

B TB treatment outcomes of cured and treatment completed are considered as successfully completed treatment.

C Row percentage calculated with total number of patients started on treatment as the denominator.

D Row percentage calculated with total number of patients successfully completed treatment as the denominator.

### Occupation and work absenteeism at start and end of treatment

Absence from work or school declined from 73% at treatment initiation to 10% at completion (Table 2). At the end of treatment, clinic visits were the main reason for absenteeism. Occupation and income remained largely unchanged.

**Table 2.** Occupation and work absenteeism in the same TB patients aged ≥18 years assessed for multimorbidity^A^ at start and end of TB treatment in selected health facilities in four African countries, June 2024 to September 2025.

Characteristics	Start of TB treatment	End of TB treatment
	n (%)	n (%)
Total	1,146	1,146
Occupation status		
Unemployed	202 (18)	182 (16)
Employed (formal)	254 (22)	277 (24)
Self-employed (own business/farm)	366 (32)	353 (31)
Daily wage labourer (informal)	235 (21)	249 (22)
Homemaker	33 (3)	30 (3)
Student	48 (4)	31 (3)
Retired	8 (1)	24 (2)
Change in income due to diagnosis/treatment of TB		
No	—	966 (85)
Yes, increased	—	86 (7)
Yes, decreased	—	94 (8)
Absent from work/school^B^		
Yes	660 (73)	93 (10)
Reason for being absent^C^		
Severity of symptoms	496 (75)	2 (2)
Visiting health facility	357 (54)	77 (83)
Doctor advice – sick leave	32 (5)	4 (4)
Patient’s fear of spreading infection	12 (2)	1 (1)
Compulsory sick leave from workplace	5 (1)	1 (1)
Other	0 (0)	12 (13)
Median (IQR) days of absenteeism before TB diagnosis at start and 1 month prior to end of treatment	7 (3–21)	2 (1–4)
Patient was the primary bread winner of the family and was absent from work^D^	360 (55)	47 (51)

IQR = interquartile range.

A Multimorbidity = comorbidities, risk factors, and disability.

B Includes those who were employed, self-employed, a daily wage labourer, and a student.

C Several patients had multiple reasons for being absent, and numbers and percentages do not add up to total absentees or 100%.

D Includes those who were employed, self-employed, and a daily wage labourer.

### Multimorbidity for the same patients at start and end of treatment

There was a major decline in prevalence of symptoms from 96% to 9% (Table 3). Comorbidities remained stable except for mental health disorder and risk factors, which decreased considerably, disability (6MWT) fell from 20% to 4%, and multimorbidity (≥3 conditions) from 20% to 8% (Table 3). The median time required to complete assessments was 30 (24–36) min.

**Table 3.** On-going symptoms, comorbidities, risk determinants, and disability of TB patients aged ≥18 years who were assessed at both start and end of TB treatment in selected health facilities in four African countries, June 2024 to September 2025.

Category	Start of TB treatment	End of TB treatment
	n (%)	n (%)
	1,146 (100)	1,146 (100)
On-going symptoms suggestive of TB		
Any symptom^A^	1,102 (96)	105 (9)
Cough	989 (86)	47 (4)
Fever	571 (50)	10 (1)
Significant weight loss	690 (60)	3 (<1)
Night sweats	633 (55)	4 (<1)
Shortness of breath	485 (42)	10 (1)
Tiredness/fatigue	600 (52)	28 (2)
Chest pain	711 (62)	16 (1)
Other^B^	125 (11)	26 (2)
Comorbidities		
Known HIV	373 (33)	389 (34)
Known DM/hyperglycaemia	46 (4)	57 (5)
Known hypertension/high blood pressure	109 (10)	119 (10)
Known mental health disorder/probable depression	77 (7)	46 (4)
Risk factors		
Probable alcohol dependence (CAGE score ≥ 2)	194 (17)	53 (5)
Malnutrition (BMI < 18.5 kg/m^2^)	435 (38)	201 (18)
Silica dust (occupational exposure)	59 (5)	62 (5)
Smoked tobacco (anytime in last 1 month)	174 (15)	107 (9)
Recreational drug use (current use)^C^	71 (6)	39 (3)
Disability		
6MWT Done	1,081 (94)	1,133 (99)
6MWT < 400 m	214 (20)^D^	43 (4)^D^
Multimorbidity (including risk factors and disability)		
None	259 (23)	434 (38)
One	367 (32)	436 (38)
Two	293 (25)	190 (17)
Three and above	227 (20)	86 (8)
Duration of assessment (in minutes)		
Median (IQR)	29 (20–37)	30 (24–36)
<15	73 (6)	90 (8)
15–29	511 (45)	383 (33)
30–44	456 (40)	598 (52)
≥45	106 (9)	75 (7)

DM = diabetes mellitus; CAGE = Cut, Annoyed, Guilty, Eye questionnaire; BMI = body mass index; 6MWT = 6-minute walk test; IQR = interquartile range.

A Some patients had multiple symptoms.

B Other symptoms include myalgia, joint pain, abdomen discomfort, and numbness of hands/feet.

C Recreational drugs included marijuana, trihexyphenidyl, and kuber (chewable nicotine).

D Percentages calculated with those in whom 6MWT was done as the denominator.

### Multimorbidity at start of TB treatment that became absent at end of treatment

As shown in Table 4, 90% of those who were symptomatic at the start of treatment were symptom-free at the end of treatment, though 4% of patients reported persistent cough. There was a substantial reduction in risk factors by the end of treatment: alcohol dependence became absent in 82%, smoking in 43%, and recreational drug use in 52%. There was a major improvement in disability: 87% of patients with disability at start of treatment were free of it by completion.

**Table 4.** Presence or absence of conditions at the end of the TB treatment amongst TB patients aged ≥18 who had these conditions documented at the start of treatment in selected health facilities in four African countries, June 2024 to September 2025.

Variable	Individuals with condition at the start of treatment	Condition at end of treatment	
		Present	Absent
		n (%)	n (%)
Symptoms			
Any symptom^A^	1,102	105 (10)	997 (90)
Cough	989	43 (4)	946 (96)
Fever	571	5 (1)	566 (99)
Significant weight loss	690	2 (<1)	688 (99)
Night sweats	633	2 (<1)	631 (99)
Shortness of breath	485	6 (1)	479 (99)
Tiredness/fatigue	600	17 (3)	583 (97)
Chest pain	711	11 (2)	700 (98)
Other^B^	125	8 (6)	117 (94)
Comorbidities			
HIV	373	373 (100)	0 (0)
DM/hyperglycaemia^C^	46	41 (89)	5 (11)
Hypertension/high blood pressure^C^	109	97 (89)	12 (11)
Mental health disorder plus new probable depression^C^	77	33 (43)	44 (57)
Risk factors			
Probable alcohol dependence (CAGE score ≥ 2)	194	34 (18)	160 (82)
Malnutrition (BMI < 18.5 kg/m^2^)	435	191 (44)	244 (56)
Occupational exposure of silica	59	58 (98)	1 (2)
Smoked tobacco (in last 1 month)	174	99 (57)	75 (43)
Current use of recreational drugs^D^	71	34 (48)	37 (52)
Disability			
Disability (6MWT < 400 m)^E^	210	28 (13)	182 (87)
Multimorbidity (including risk factors and disability)			
One or more	887	685 (77)	202 (23)

DM = diabetes mellitus; CAGE = Cut, Annoyed, Guilty, Eye questionnaire; BMI = body mass index; 6MWT = 6-minute walk test.

A Some patients had multiple symptoms.

B Other symptoms include myalgia, joint pain, abdomen discomfort, and numbness of hands/feet.

C Individuals with hyperglycaemia, high blood pressure, and probable depression were not necessarily confirmed to have DM, hypertension, or mental health disorders, respectively.

D Recreational drugs included marijuana, trihexyphenidyl, and kuber (chewable nicotine).

E The total (210) includes those in who 6MWT was done at start of treatment and 6MWT was less than 400 meters. Percentages calculated with those in whom 6MWT was done as the denominator.

### Multimorbidity at end of treatment which was absent at start of treatment

Although numbers were small (between 1% and 2% of all cases), new cases of comorbidities, risk factors, and disability were reported or detected at the end of treatment (Table 5).

**Table 5.** Presence or absence of conditions at the end of the TB treatment amongst TB patients aged ≥18 without these conditions at the start of treatment in selected health facilities in four African countries, June 2024 to September 2025.

Variable	Individuals without condition at the start of treatment	Condition at end of treatment	
		Present	Absent
		n (%)^A^	n (%)^A^
Symptoms			
Any symptom^B^	44	0 (0)	44 (100)
Cough	157	4 (3)	153 (97)
Fever	575	5 (1)	570 (99)
Significant weight loss	456	1 (<1)	455 (99)
Night sweat	513	2 (<1)	511 (99)
Shortness of breath	661	4 (1)	657 (99)
Tiredness/fatigue	546	11 (2)	535 (98)
Chest pain	435	5 (1)	430 (99)
Other^C^	1,021	18 (2)	1,003 (98)
Comorbidities			
HIV	773	16 (2)	757 (98)
DM/hyperglycaemia	1,100	16 (1)	1,084 (99)
Hypertension/high blood pressure	1,037	22 (2)	1,015 (98)
Mental health disorder plus new probable depression	1,069	13 (1)	1,056 (99)
Risk factors			
Probable alcohol dependence (CAGE score ≥ 2)	952	19 (2)	933 (98)
Malnutrition (BMI < 18.5 kg/m^2^)	711	10 (1)	701 (99)
Occupational exposure of silica	1,087	4 (<1)	1,083 (99)
Smoked tobacco (in last 1 month)	972	8 (1)	964 (99)
Current use of recreational drugs^D^	1,075	5 (<1)	1,070 (99)
Disability			
Disability (6MWT <400 m)^E^	923	15 (2)	908 (98)
Multimorbidity			
One or more	259	27 (10)	232 (90)

DM = diabetes mellitus; CAGE = Cut, Annoyed, Guilty, Eye questionnaire; BMI = body mass index; 6MWT = 6-minute walk test.

A Percentage calculated with individuals without condition at the start of treatment as the denominator.

B Some patients had multiple symptoms.

C Other symptoms such as myalgia, joint pain, abdomen discomfort, and numbness of hands/feet.

D Recreational drugs included marijuana, trihexyphenidyl, and kuber (chewable nicotine).

E The total (923) includes those in whom 6MWT was not done during start of treatment and those in whom it was done and 6MWT was more than or equal to 400.

### Characteristics associated with disability at the end of treatment

Disability was independently associated with female sex (aPR 2.7, 95% CI: 1.5–4.7), mental health disorder (aPR 2.9, 95% CI: 1.2–7.2), probable alcohol dependence (aPR 3.2, 95% CI: 1.4–0.4), and malnutrition (aPR 2.9, 95% CI: 1.4–5.9) at the end of treatment (Supplementary Data Table S1).

### Referral for care

In general, referral for care was >85% except for silica exposure (23%), smoking (57%), and recreational drug use (46%). Referrals for most multimorbidities were within-facility, except for silica exposure and disability (∼35%) (see Supplementary Data Table S2).

## DISCUSSION

This is the first multi-country implementation research study conducted within African NTPs that demonstrates the benefits of offering a package of early screening, referral, and comprehensive care for multimorbidity during TB treatment. Work and school absenteeism declined sharply, and health outcomes improved with marked reductions in symptoms, disability, and risk factors. Referral systems generally functioned well, though gaps remained for silica exposure and disability. These findings have important implications for public health. They show that efforts to achieve a ‘fourth 90’^22^ (ensuring that at least 90% of people with TB attain good health-related quality of life which complements the 2014 Stop TB Partnership 90-[90]-90 targets) can produce tangible outcomes. This evidence can also guide the implementation of the recent WHO policy brief on addressing TB-associated disability^7^ by demonstrating approaches on ‘how to’ implement.

Three key findings have important policy and practice implications:

First, in a previous cohort from the same four African countries – where multimorbidity was assessed only at the end of TB treatment^13^ – the prevalence of persistent symptoms (26%) and disability (17%) was higher than in the current cohort, which benefited from early assessment and care soon after treatment initiation. The current cohort showed markedly better outcomes, including sharp reductions in multimorbidity and an 80% decline in TB-related disability. Although the exact reasons remain unclear, early screening and timely referrals likely played a key role. Previous work showed that TB-associated lung function is impaired early in the course of treatment suggesting opportunities for early intervention.^23^ Studies in different countries have recently shown that low-cost pulmonary rehabilitation programmes in TB survivors can lead to better symptom management, increased exercise capacity, improved health, and better quality of life.^24,25^ It is plausible that the observed improvements in our study were related to referrals for multimorbidity management and pulmonary rehabilitation services where available.

Second, although the frequency of various types of multimorbidity decreased at the end of treatment, a proportion of patients continued to engage in harmful behaviours such as alcohol use, smoking, and recreational drug consumption. In addition, a small number of patients took up these behaviours during treatment, possibly due to improved health and return to previous habits. Such behaviours heighten the risk of recurrent TB and increased mortality after treatment completion.^26,27^ On-going comorbidities in this cohort, including HIV infection, DM, and hypertension, further compound these risks.^28,29^ This evidence underscores the need for systematic assessment and care at both start and end of treatment, and beyond, to mitigate these behaviours and manage comorbidities to reduce recurrent TB and promote long-term survival.

Third, in contrast to a previous study,^30^ it was encouraging to observe that absenteeism from work or school had declined to 10% by the end of treatment, with most of this absence attributable to visiting health facilities for TB medicine collection, submission of sputum specimens, and assessment for multimorbidity. Levels of income remained largely unchanged during TB treatment. However, we did not assess for expenses that can contribute to catastrophic costs during TB treatment.

There were several study strengths. It is programmatically relevant, embedded within the NTP, and addresses an identified research priority, both at national and international level.^7^ We have also demonstrated synergies between the TB programme and general health services.^31^ Data were collected in real-time with regular quality assurance. The study followed the Strengthening the Reporting of Observational Studies in Epidemiology (STROBE) guidelines.^32^ However, there were some limitations. In both Uganda and Zambia, a smaller proportion of patients who successfully completed treatment (72% and 53%, respectively) were assessed for multimorbidity, which may have affected the overall representativeness of the outcome measures. This coincided with the reduction in USAID funding in early 2025, which impacted several NTPs through job lay-offs, disruptions in monitoring, evaluation, and supervision, as well as reduced operational support from implementing partners.^33^ In Zimbabwe, we did not reach the target sample size during recruitment because of lower patient numbers in health facilities. Additionally, we lacked data on the type, duration, and quality of care received after referrals following administered care. Despite these limitations, our findings have important policy and practice implications. We have demonstrated that conducting multimorbidity assessments at both the start and end of TB treatment is feasible and valuable. By identifying and addressing key comorbidities, risk factors, and disability, we can improve health outcomes, potentially reduce the risk of recurrent TB, and enhance long-term survival. Importantly, this can be done within existing health services, although there is a need to accelerate the integration of multimorbidity management to all levels of the health system.

Some gaps remain. To date, our studies have focused only on adults, despite evidence that children may experience stunted growth and respiratory disorders.^34^ This highlights the need for similar operational research in children. Additionally, the critical need for low-cost, community-based pulmonary rehabilitation programmes must be addressed as evidence demonstrates their effectiveness.^24,25^ We found that women and those with mental health disorders, alcohol dependence, or malnutrition faced higher disability risk and should be prioritised for pulmonary rehabilitation where resources are limited. Finally, more resources need to be directed to providing patient-centred holistic care. National governments and major funders, including the Global Fund and others, could help bridge these gaps by incorporating these interventions into their funding streams to ensure effective implementation.^35^

## CONCLUSION

Implementing early interventions for multimorbidity at the start of TB treatment across four African countries led to marked reductions in symptoms, risk factors, and disability amongst patients with TB. These findings underscore the importance of integrated, patient-centred care at the start, during, and end of TB treatment. Our multi-country study provides a promising roadmap towards achieving this goal.

## Supplementary Material




