# Supplementary material for: Early interventions reduce multimorbidity and TB disability in Kenya, Uganda, Zambia, and Zimbabwe
Source: IJTLD Open. 2026 Mar 13;3(3):177–84. doi: 10.5588/ijtldopen.25.0656 (PMC12991729; doi:10.5588/ijtldopen.25.0656)
Supplement: Supplementary file 1 [file ijtldopen25-0656_supplementarydata1.pdf]

Supplementary Table: Characteristics associated with disability (6MWT <400 metres) at the end of treatment in TB patients aged ≥18 years in selected health facilities in four African countries, June 2024 to September 2025

**Variable**[1]	**Total**	**6MWT <400m**		**Crude**		**Adjusted**	
	**n**	**n**	**(%)**	**PR**	**(95% CI)**	**aPR**	**(95% CI)**[3]
**Total**	1133[2]	43	(3.8)				
**Age in years**							
18-29	350	10	(2.9)	1		1	
30-44	521	20	(3.8)	1.3	(0.6-2.8)	1.7	(0.8-3.7)
45-59	218	11	(5.1)	1.8	(0.8-4.1)	1.8	(0.8-4.3)
≥60	44	2	(4.6)	1.6	(0.4-7.0)	1.9	(0.4-9.4)
**Sex**							
Male	801	24	(3.0)	1		1	
Female	322	19	(5.7)	**1.9**	**(1.1-3.4)**	**2.7**	**(1.5-4.7)**
**Site of TB**							
Pulmonary	1081	39	(3.6)	0.5	(0.2-1.3)		
Extrapulmonary	52	4	(7.7)	1			
**Type of TB**							
Bacteriologically confirmed	726	31	(4.3)	1			
Clinically diagnosed	407	12	(3.0)	0.7	(0.4-1.3)		
**Category of TB**							
New	1017	40	(3.9)	1			
Previously treated	116	3	(2.6)	0.7	(0.2-2.1)		
**Drug-susceptibility**							
Sensitive	1118	42	(3.8)	1			
Resistant	15	1	(6.7)	1.8	(0.3-12.1)		
**HIV status**							
Positive	747	24	(3.2)	1			
Negative	386	19	(4.9)	1.5	(0.8-2.8)		
**Diabetes Mellitus/ hyperglycemia**							
Yes	55	2	(3.6)	1.0	(0.2-3.9)		
No	1078	41	(3.8)	1			
**High blood pressure**							
Yes	116	7	(6.0)	1.7	(0.8-3.7)		
No	1017	36	(3.5)	1			
**Mental health disorder**							
Yes	45	7	(15.6)	**4.7**	**(2.2-10.0)**	**2.9**	**(1.2-7.2)**
No	1088	36	(3.3)	1		1	
**Probable alcohol Dependence**							
Yes	51	6	(11.8)	**3.4**	**(1.5-7.8)**	**3.2**	**(1.4-7.4)**
No	1082	37	(3.4)	1		1	
**Malnutrition**							
Yes	200	17	(8.5)	**3.1**	**(1.7-5.5)**	**2.9**	**(1.4-5.9)**
No	933	26	(2.8)	1		1	
**Occupational exposure to silica**							
Yes	60	3	(5.0)	1.3	(0.4-4.2)		
No	1073	40	(3.7)	1			
**Smoked tobacco**							
Yes	104	6	(5.8)	1.6	(0.7-3.7)		
No	1029	37	(3.6)	1			
**Recreational drug use**							
Yes	39	5	(12.8)	3.7	(1.5-8.9)	2.2	(0.8-6.3)
No	1094	38	(3.5)	1		1	
**Multimorbidity (excluding 6MWT)**							

None	431	7	(1.6)	1		1	
One	438	14	(3.2)	2	(0.8-4.9)	1.3	(0.5-3.4)
Two	186	14	(7.5)	4.7	(1.9-11.5)	1.9	(0.7-5.4)
Three and above	71	8	(11.3)	7.1	(2.6-18.8)	1.4	(0.4-5.4)

[1] The comorbidities and risk determinants were based on the end of treatment assessment

[2] 1133 patients underwent the 6MWT

[3] Only age and other variables which were significant in the univariate analysis were included in the multivariate model. The multi-collinearity was assessed using variance inflation factor and there was no multi-collinearity in variables included in the model

Abbreviations: PR- Prevalence ratio, aPR- Adjusted prevalence ratio; TB- Tuberculosis; HIV- Human Immunodeficiency Virus; 6MWT- Six Minute Walk Test; CI- Confidence Interval

Supplementary Table 2: Referral for further care in TB patients aged ≥18 years with multimorbidity at the end of treatment in selected health facilities in four African countries, June 2024 to September 2025

**Conditions**	**Eligible for referral[1]**	**Referred to care**		**Referred within the same facility**	
	**n**	**n**	**(%)**	**n**	**(%)[2]**
**Comorbidities**					
Diabetes Mellitus/ hyperglycemia	15	13	(87)	13	(100)
Hypertension	23	23	(100)	43	(100)
HIV[3]	1	1	(100)	1	(100)
Mental health disorder	15	15	(100)	16	(100)
**Risk factors**					
Probable alcohol dependence	53	48	(91)	48	(100)
Malnutrition	201	182	(91)	178	(98)
Occupational exposure to silica	62	14	(23)	5	(36)
Smoking	107	61	(57)	61	(100)
Recreational drug use	39	18	(46)	18	(100)
**Disability**					
6MWT <400 metres	43	37	(86)	12	(32)

Multimorbidity = Comorbidities, risk factors and disability

[1]For diabetes mellitus, hypertension and mental health disorder, patients who were newly diagnosed and patients who were known to have the condition but not on care were considered eligible for referral

[2]Percentages calculated with total referred for the condition as denominator

[3]Of the 16 patients whose HIV status emerged by the end of treatment, 15 were already on antiretroviral therapy and only 1 needed referral for antiretroviral therapy

Abbreviation: TB- Tuberculosis; HIV- Human Immunodeficiency Virus; 6MWT- Six Minute Walk Test
